# Using Shear Wave Elastography to Predict Medical Treatment Response in Tubo-Ovarian Abscess

**DOI:** 10.3390/biomedicines14081671

**Published:** 2026-07-24

**Authors:** Serav Koç, Şule Gül Aydın, Hasan Can Toyganözü, Emel Lüftüoğlu, Ali Can Koç, Cenk Parlatan, Cevdet Adıgüzel, Emre Destegül

**Affiliations:** 1 Department of Obstetrics and Gynecology, Yüksekova State Hospital, 30300 Hakkari, Turkey; 2 Department of Obstetrics and Gynecology, Adana City Training and Research Hospital, 01170 Adana, Turkey; sulegulaydin@gmail.com (Ş.G.A.);; 3 Department of Obstetrics and Gynecology, Private Medline Adana Hospital, 01170 Adana, Turkey; hasanbaskent@hotmail.com; 4 Department of Obstetrics and Gynecology, Elazığ Fethi Sekin City Hospital, 23280 Elazığ, Turkey; 5 Department of Radiology, Baskent University Adana Dr. Turgut Noyan Application and Research Center, 01250 Adana, Turkey; 6 Department of Obstetrics and Gynecology, Medical Park Adana Hospital, 01060 Adana, Turkey; cevdetadiguzel@yahoo.com; 7 Department of Obstetrics and Gynecology, Private Adana Middle East Hospital, 01140 Adana, Turkey; destegulemre@gmail.com

**Keywords:** pelvic inflammatory disease, tubo-ovarian abscess, shear wave elastography

## Abstract

**Background**: This study aimed to evaluate the clinical utility of shear wave elastography (SWE) in assessing abscess wall stiffness and its potential contribution to predicting the need for surgical intervention in patients with tubo-ovarian abscess (TOA). **Materials and methods**: This prospective cross-sectional study included 34 women diagnosed with TOA who underwent SWE evaluation upon admission. Demographic characteristics, laboratory parameters, abscess size, abscess wall elasticity, and abscess content elasticity were recorded. These patients were categorized as either medically managed or surgically treated. Group comparisons, receiver operating characteristic (ROC) curve analysis, and multivariable binary logistic regression were performed to identify independent predictors of surgical intervention. **Results**: There were no significant differences between the operated and non-operated groups regarding age, body mass index, and laboratory parameters (*p* > 0.05). The median length of hospital stay was significantly longer in the operated group (15 vs. 12 days, *p* = 0.017). Abscess diameter was significantly larger in the operated group (6.75 ± 1.17 cm vs. 4.65 ± 1.20 cm; *p* = 0.001). Likewise, the median abscess wall SWE value was significantly higher in the operated group (3.12 vs. 1.47 kPa; *p* = 0.027). The optimal cut-off value for abscess wall SWE was 2.04 kPa (AUC: 0.721, 95% CI: 0.551–0.892, sensitivity: 64.7%, specificity: 70.6%). Abscess size demonstrated a higher predictive performance (AUC: 0.905, 95% CI: 0.796–1.000). However, in the multivariable logistic regression analysis, only abscess size remained an independent predictor of surgical intervention (OR: 4.14, 95% CI: 1.50–11.39, *p* = 0.006), whereas abscess wall SWE and baseline C-reactive protein were not independently associated with treatment failure. **Conclusions**: Increased abscess wall stiffness measured by SWE was associated with the need for surgical intervention in patients with TOA. Although SWE was not an independent predictor after adjustment for abscess size and baseline C-reactive protein, it may serve as a complementary imaging biomarker when interpreted together with established clinical, laboratory, and conventional ultrasonographic findings.

## 1. Introduction

Pelvic inflammatory disease (PID) is defined as an infection of the upper female genital tract and typically results from the ascending spread of pathogens originating in the lower genital tract [1]. PID encompasses a broad spectrum of infections involving the upper genital structures such as salpingitis, endometritis, and peritonitis, and in advanced cases may progress to the formation of pelvic abscesses. Tubo-ovarian abscess (TOA) is considered one of the most severe complications of PID and, if left untreated, may lead to sepsis, peritonitis, and multi-organ failure, resulting in substantial morbidity and mortality [2]. Despite appropriate treatment, TOA develops in approximately 15–34% of patients diagnosed with PID [3]. TOA and pelvic abscesses are typically characterized by an inflammatory mass involving the ovaries, fallopian tubes, and/or adjacent pelvic tissues [4].

Abdominal adhesions developing after PID or TOA may result in long-term complications such as infertility, ectopic pregnancy, and chronic pelvic pain. These adhesions disrupt the normal anatomical relationships of pelvic organs, adversely affecting reproductive function [5].

Ultrasound elastography (USE) is a non-invasive imaging technique that assesses tissue elasticity or stiffness based on the characteristics of acoustic waves as they propagate through biological tissues [6,7]. In this technique, the acoustic force generated by the transducer induces tissue displacement, producing shear waves that travel perpendicular to the applied force. The velocity of these shear waves is measured quantitatively and converted into Young’s modulus, expressed in kilopascals (kPa) [7]. Numerous studies have demonstrated the clinical utility of shear wave elastography (SWE) in applications such as liver fibrosis staging and characterization of breast lesions [8,9]. The method’s high reproducibility and ease of use have further increased its popularity in clinical and research settings.

In this study, SWE was performed in patients diagnosed with TOA in our clinic to evaluate its ability to predict the response to medical therapy. The aim was to investigate whether SWE can serve as a useful imaging modality for assessing treatment success in TOA and to provide scientific support for its potential integration into clinical management. This study represents one of the first investigations evaluating SWE findings as predictors of medical treatment response in TOA.

## 2. Materials and Methods

This prospective cross-sectional study was conducted at the Obstetrics and Gynecology Clinic of the Health Sciences University Adana City Training and Research Hospital between February and June 2025. The study included 34 women who were hospitalized with a diagnosis of TOA and who underwent SWE to evaluate treatment response. Ethical approval was obtained from the Clinical Research Ethics Committee of Adana City Training and Research Hospital (date: 6 February 2025; Decision No.: 343), and written informed consent was obtained from all participants prior to study enrollment.

Women aged 18 years or older who were admitted with a diagnosis of tubo-ovarian abscess (TOA), had an abscess measuring ≤9 cm on initial ultrasonographic evaluation, and whose abscess location and depth were suitable for SWE assessment were eligible for inclusion. Patients who refused to participate, required urgent surgery before SWE could be performed because of an acute abdomen, were pregnant, had a known or newly diagnosed malignancy, or had an abscess measuring >9 cm were excluded. Of the initial 37 women diagnosed with TOA, three were excluded because a malignancy was identified during the diagnostic work-up.

Demographic and clinical variables, including age, body mass index (BMI), and history of previous abdominal surgery, were recorded. Length of hospitalization (days) and laboratory parameters—including hemoglobin (Hb), white blood cell count (WBC), platelet count (PLT), C-reactive protein (CRP), and procalcitonin (PCT)—were monitored during follow-up. All patients were evaluated by the same radiologist using the same device as soon as possible after admission. In the majority of patients, SWE examinations were performed before the initiation of intravenous antibiotic therapy. However, a small number of patients referred from other healthcare centers had received up to two doses of intravenous antibiotics prior to imaging.

Treatment decisions were made according to the institutional management protocol and current clinical practice. Surgical intervention was considered to represent medical treatment failure and was performed in patients who demonstrated persistent fever despite 48–72 h of appropriate intravenous antibiotic therapy, persistent elevation or lack of improvement in inflammatory markers (particularly C-reactive protein [CRP] and procalcitonin levels and white blood cell count [WBC]), worsening abdominal pain despite treatment, increasing abscess size on follow-up imaging, suspected abscess rupture, generalized peritonitis, sepsis, or hemodynamic instability, or when the attending multidisciplinary team considered that conservative management was unlikely to succeed based on the patient’s overall clinical, laboratory, and imaging findings.

All ultrasonographic assessments, including SWE, were performed using a Philips EPIQ 7 ultrasound system equipped with a C5-1 high-resolution convex probe (Philips Healthcare, Bothell, WA, USA). All SWE examinations were performed using the manufacturer’s standard two-dimensional ElastQ shear wave elastography preset. Acoustic radiation force impulses were generated automatically by the system using the default acoustic output settings. No manual adjustments were made to the acoustic output parameters during image acquisition. All examinations were performed within established diagnostic ultrasound safety limits (Mechanical Index < 1.9 and derated ISPTA.3 < 720 mW/cm^2^), in accordance with international regulatory recommendations. The C5-1 convex transducer was used within its standard abdominal frequency range of 1–5 MHz. The same preset, gain, depth, focus, and elastography settings were maintained for all patients. The pulse repetition frequency and frame rate were automatically optimized by the device according to lesion depth and tissue characteristics and were not manually modified during the examinations.

Patients were positioned to obtain optimal lesion visualization (supine or lateral decubitus), and sufficient gel was applied to eliminate potential artifacts. Abscess formation was first assessed in B-mode to determine the anteroposterior and mediolateral diameters. After dimensional evaluation, SWE measurements were performed by placing a rectangular region of interest (ROI) on the thickest visually continuous portion of the abscess wall while avoiding visible septations, cystic or necrotic areas, calcifications, major vessels, and regions affected by acoustic shadowing or motion artifacts. Care was taken to position the ROI entirely within the abscess wall without including adjacent pelvic tissues. Subsequently, SWE measurements were obtained from the abscess content using the same standardized approach whenever technically feasible. All ROIs were positioned perpendicular to the anterior margin, with a maximum ROI depth of 8 cm and an average ROI size of 1 cm. Measurements were performed in the axial plane, and elasticity values were recorded in kilopascals (kPa). To minimize measurement variability, minimal transducer compression was applied during image acquisition, and all measurements were obtained after a stable elastography color map had been achieved. All data were stored digitally. The radiologist performing the examinations had eight years of professional experience and performs approximately 100 SWE procedures annually. Each assessment session lasted approximately 10–15 min.

Statistical analyses were conducted using IBM SPSS Statistics version 26.0 (IBM Corp., Armonk, NY, USA). Continuous variables are presented as the mean ± standard deviation or median (minimum–maximum), while categorical variables are expressed as numbers and percentages. The Shapiro–Wilk test was used to assess normality. For group comparisons, the independent samples *t*-test was applied to normally distributed variables, whereas the Mann–Whitney U test was used for non-normally distributed variables. Chi-square testing was used for categorical comparisons. Receiver operating characteristic (ROC) curve analysis was performed to determine the optimal cut-off values for predicting treatment response. Variables considered clinically relevant (baseline C-reactive protein [CRP], abscess size, and abscess wall SWE) were entered into a multivariable binary logistic regression model using the enter method to identify independent predictors of surgical intervention (medical treatment failure). Odds ratios (ORs) with 95% confidence intervals (CIs) were calculated. A *p*-value < 0.05 was considered statistically significant.

## 3. Results

Thirty-four patients diagnosed with TOA were evaluated. The demographic characteristics and laboratory findings of the patients are summarized in Table 1. No statistically significant differences were found between the operated and non-operated groups in terms of age, BMI, previous abdominal surgery, and laboratory findings (*p* > 0.05). The median length of hospital stay was 15 [12–28] days in the operated group and 12 [10–15] days in the non-operated group. A statistically significant difference in hospitalization duration was observed between the groups (*p* = 0.017).

Abscess size and SWE measurements were compared between the groups (Table 2). The mean abscess diameter and median abscess wall SWE value were significantly higher in the operated group compared to the non-operated group (6.75 ± 1.17 cm vs. 4.65 ± 1.20 cm, *p* = 0.001; 3.12 [1.26–6.49] kPa vs. 1.47 [0.86–3.15] kPa, *p* = 0.027). The median abscess content SWE value was also higher in the operated group than in the non-operated group, but the difference was not statistically significant (1.11 [0.85–4.03] kPa vs. 0.90 [0.63–1.18] kPa, *p* = 0.071).

**Figure 1:**
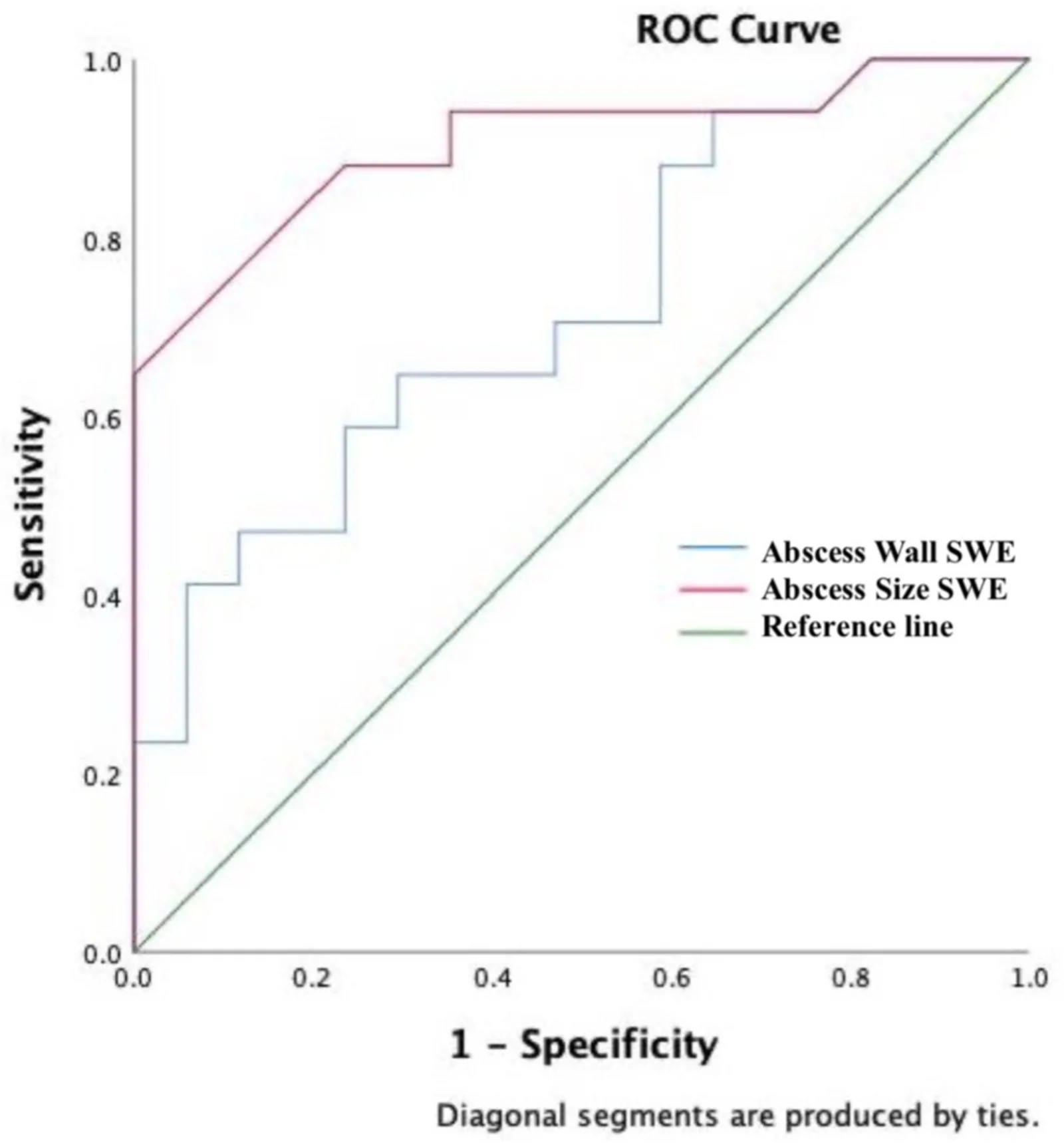
ROC curve.

ROC analysis was performed to evaluate the predictive performance of abscess wall shear wave elastography (SWE) and abscess size for identifying patients requiring surgical intervention (Table 3). The optimal cut-off value was 2.04 kPa for abscess wall SWE and 5.5 cm for abscess size. The area under the curve (AUC) was 0.721 (95% CI: 0.551–0.892, *p* = 0.012) for abscess wall SWE and 0.905 (95% CI: 0.796–1.000, *p* < 0.001) for abscess size. Using these thresholds, abscess wall SWE demonstrated a sensitivity of 64.7% and specificity of 70.6%, whereas abscess size showed a sensitivity of 88.2% and specificity of 76.5% for predicting the need for surgical intervention (Figure 1).

A multivariable binary logistic regression analysis was subsequently performed to identify independent predictors of surgical intervention (Table 4). Baseline CRP, abscess size, and abscess wall SWE values were included in the model. Among these variables, only abscess size remained independently associated with the need for surgical intervention (OR: 4.14, 95% CI: 1.50–11.39, *p* = 0.006). Neither baseline CRP (*p* = 0.332) nor abscess wall SWE (*p* = 0.537) remained significant independent predictors after adjustment.

## 4. Discussion

In recent years, there has been a shift away from aggressive surgical approaches in the management of TOA, with an increasing preference for medical and minimally invasive strategies, as highlighted in several publications [10,11,12,13]. The literature similarly emphasizes the high success rates of conservative methods such as ultrasound- or CT-guided drainage and their ability to reduce unnecessary surgical interventions [10]. However, there is still no standardized set of criteria to determine which patients require surgery. Therefore, the need for new predictive markers remains. In our study, the median abscess wall SWE value was significantly higher in the operated group (3.12 [1.26–6.49] kPa) compared with the non-operated group (1.47 [0.86–3.15] kPa) (*p* = 0.027). The optimal cut-off for abscess wall SWE was 2.04 kPa, with an area under the curve (AUC) of 0.721 (*p* < 0.012). At this threshold, abscess wall SWE predicted the need for surgery with a sensitivity of 64.7% and specificity of 70.6%. However, multivariable binary logistic regression analysis demonstrated that only abscess size remained independently associated with the need for surgical intervention, whereas abscess wall SWE did not remain an independent predictor after adjustment for baseline CRP and abscess size. These findings suggest that SWE should be considered a complementary imaging biomarker rather than a stand-alone predictor of medical treatment failure.

Recent studies have emphasized the substantial potential of SWE in the diagnosis of inflammatory diseases, given its ability to objectively quantify tissue stiffness. In acute surgical conditions such as appendicitis, elevated elasticity modulus values obtained through SWE have been shown to correlate with the presence of inflammation. Notably, a threshold value of 12.5 kPa was reported to yield 100% specificity in SWE-based evaluation of the appendix, demonstrating that this technique provides a reliable biomechanical parameter for detecting inflammatory processes [14]. These findings are consistent with the results of our study where increased abscess wall stiffness was associated with the need for surgical intervention in the univariable analysis. Although this association did not persist after adjustment for abscess size and baseline CRP, SWE may still provide valuable complementary information regarding the severity of the inflammatory process. Increased wall stiffness may reflect a more organized and treatment-resistant inflammatory response, supporting the potential role of SWE as an adjunct to conventional clinical and ultrasonographic assessment rather than as an independent predictor of treatment failure. Increased wall stiffness appears to reflect a more severe and resistant inflammatory process, thereby indicating a higher likelihood of requiring surgical intervention. Accordingly, it may be concluded that clinical decision-making should incorporate not only morphological characteristics, but also biomechanical parameters that capture tissue stiffness. SWE thus holds value as a complementary tool for assessing the severity of inflammation and guiding the selection of appropriate treatment strategies.

Destegül et al. demonstrated that shear wave elastography could predict the depth of myometrial invasion in endometrial cancer with good diagnostic performance, supporting its potential as a non-invasive tool for preoperative assessment [15]. These findings suggest that SWE may provide clinically relevant information beyond conventional morphological imaging by reflecting underlying tissue characteristics. Nevertheless, further studies are required to better define its role in clinical decision-making.

Şirinoğlu et al. reported increased placental stiffness in patients with gestational diabetes mellitus, suggesting that SWE may serve as a non-invasive and quantitative imaging tool for assessing tissue changes associated with inflammatory and fibrotic processes. However, further studies are needed to validate its clinical utility [16].

SWE has also been increasingly applied in the evaluation of breast lesions. Although it improves diagnostic performance when combined with conventional ultrasound, variability between devices and tumor heterogeneity require elastography findings to be interpreted alongside morphological imaging rather than as a stand-alone diagnostic tool [17].

Beyond breast imaging, SWE has been increasingly applied to the thyroid, liver, and gastrointestinal system [18], and its utility in infectious conditions, particularly in distinguishing fibrotic from necrotic tissue within abscesses, has also been demonstrated. It should be noted that the absolute SWE values observed in the present study were lower than those reported for certain other inflammatory conditions, such as acute appendicitis. This difference is not unexpected, as SWE measurements are influenced by tissue composition, anatomical location, lesion depth, and technical factors related to ultrasound acquisition. Tubo-ovarian abscesses are complex lesions containing variable proportions of purulent fluid, necrotic material, inflammatory tissue, and surrounding adnexal structures, all of which may affect tissue stiffness measurements. In addition, the deep pelvic location of TOAs may reduce shear wave propagation and measurement stability compared with more superficial organs. Therefore, absolute SWE values should not be directly compared across different organs and disease entities but should instead be interpreted within the specific clinical context of tubo-ovarian abscess. In our study, SWE values were found to be more informative when interpreted in conjunction with clinical parameters such as abscess diameter and length of hospitalization, suggesting that multifactorial assessment enhances the ability to predict medical treatment success.

This study has several limitations. First, the relatively small sample size and single-center design may have limited the statistical power and external validity of our findings. In addition, the multivariable logistic regression analysis was based on only 17 surgical events. Although the number of variables included in the model was intentionally restricted to minimize the risk of overfitting, the limited number of events may still have affected the stability of the regression estimates. Therefore, the multivariable findings should be interpreted with caution. Thus, the results should be considered preliminary and require confirmation in larger prospective multicenter studies. Second, SWE measurements were performed using a standardized acquisition protocol by a single experienced radiologist and, therefore, interobserver and intraobserver reproducibility were not evaluated. Consequently, operator-related variability could not be assessed. Finally, although the SWE examinations were performed before antibiotic therapy in the vast majority of patients, a small number of participants had received up to two doses of intravenous antibiotics prior to imaging. Early antibiotic treatment may have partially modified the inflammatory process and tissue stiffness, thereby contributing to variability in elastographic measurements. Although this effect is likely to have been limited, it cannot be excluded and should be considered when interpreting the findings. Future studies should include reproducibility analyses and larger multicenter cohorts to further establish the clinical utility of SWE in patients with TOA.

## 5. Conclusions

In conclusion, increased abscess wall stiffness measured by shear wave elastography was associated with a higher likelihood of surgical intervention in patients with tubo-ovarian abscess. However, after adjustment for abscess size and baseline C-reactive protein, SWE was not an independent predictor of treatment failure. Therefore, SWE may serve as a complementary imaging biomarker when interpreted together with established clinical, laboratory, and conventional ultrasonographic findings, particularly abscess size, rather than as a stand-alone predictor. The proposed SWE cut-off value of 2.04 kPa should be considered exploratory and should not be used in routine clinical practice until validated in larger prospective multicenter studies and independent external cohorts.

## Figures and Tables

**Table 1:** Demographic characteristics and laboratory findings of the participants.

Variable	Operated (*n* = 17)	Non-Operated (*n* = 17)	*p*-Value
Age (years)	36.47 ± 9.13	39.58 ± 8.93	0.322
BMI (kg/m^2^)	25.48 ± 3.75	26.51 ± 3.91	0.440
History of abdominal surgery, n (%)			0.640
(+)	11 (64.7%) ^a^	11 (64.7%) ^a^	
(−)	6 (35.3%) ^a^	6 (35.3%) ^a^	
Hb (g/dL)	11.06 ± 1.84	10.81 ± 1.80	0.695
WBC	16.84 ± 5.84	14.5 ± 5.01	0.226
Platelets (10^3^/μL)	382.41 ± 148.87	392.8 ± 158.64	0.844
CRP	167.93 ± 96.73	166.66 ± 111.82	0.970
Procalcitonin	0.23 [0.05–1.88]	0.1 [0.03–0.28]	0.202
Length of hospital stay (days)	15 [12–28]	12 [10–15]	0.017

Abbreviations: BMI: body mass index; Hb: hemoglobin; WBC: white blood cell count; CRP: C-reactive protein. ^a^ Chi-square test.

**Table 2:** Abscess size and sonoelastography findings of the participants.

Variable	Operated (*n* = 17)	Non-Operated (*n* = 17)	*p*-Value
Abscess Size (cm)	6.75 ± 1.17	4.65 ± 1.20	0.001
Abscess Wall Elastography (kPa)	3.12 [1.26–6.49]	1.47 [0.86–3.15]	0.027
Abscess Content Elastography (kPa)	1.11 [0.85–4.03]	0.9 [0.63–1.18]	0.071

Abbreviations: cm: centimeter; kPa: kilopascal.

**Table 3:** Predictive performance of shear wave elastography parameters for determining surgical intervention in patients with TOA.

Variable	Cut-Off Value	AUC (95% CI)	*p*-Value	Sensitivity (%)	Specificity (%)
Abscess wall SWE (kPa)	2.04	0.721 (0.551–0.892)	0.012	64.7	70.6
Abscess size (cm)	5.5	0.905 (0.796–1.000)	0.001	88.2	76.5

Abbreviations: TOA: tubo-ovarian abscess; SWE: shear wave elastography; AUC: area under the curve; kPa: kilopascal.

**Table 4:** Multivariable binary logistic regression analysis of predictors of surgical intervention in patients with TOA.

Variable	OR	95% CI	*p*
Baseline CRP	0.995	0.984–1.006	0.332
Abscess size (cm)	4.135	1.502–11.386	0.006
Abscess wall SWE (kPa)	1.141	0.750–1.737	0.537

Abbreviations: OR, odds ratio; CI, confidence interval; CRP, C-reactive protein; SWE, shear wave elastography.

## Data Availability

The datasets used and analyzed in the current study are available from the corresponding author upon reasonable request.

## References

[1] Jenkins S.M., Vadakekut E.S. (2025). Pelvic Inflammatory Disease. StatPearls.

[2] Kairys N., Roepke C. (2025). Tubo-Ovarian Abscess. StatPearls.

[3] Tang H., Zhou H., Zhang R. (2022). Antibiotic Resistance and Mechanisms of Pathogenic Bacteria in Tubo-Ovarian Abscess. Front. Cell. Infect. Microbiol..

[4] Granberg S., Gjelland K., Ekerhovd E. (2009). The management of pelvic abscess. Best Pract. Res. Clin. Obstet. Gynaecol..

[5] Zhu S., Ballard E., Khalil A., Baartz D., Amoako A., Tanaka K. (2021). Impact of early surgical management on tubo-ovarian abscesses. J. Obstet. Gynaecol..

[6] Brunelli A.C., Brito L.G.O., Moro F.A.S., Jales R.M., Yela D.A., Benetti-Pinto C.L. (2023). Ultrasound Elastography for the Diagnosis of Endometriosis and Adenomyosis: A Systematic Review with Meta-analysis. Ultrasound Med. Biol..

[7] Shiina T., Nightingale K.R., Palmeri M.L., Hall T.J., Bamber J.C., Barr R.G., Castera L., Choi B.I., Chou Y.H., Cosgrove D. (2015). WFUMB guidelines and recommendations for clinical use of ultrasound elastography: Part 1: Basic principles and terminology. Ultrasound Med. Biol..

[8] Ozturk A., Olson M.C., Samir A.E., Venkatesh S.K. (2022). Liver fibrosis assessment: MR and US elastography. Abdom. Radiol..

[9] Tay I.W.M., Sim L.S., Moey T.H.L., Tan K.P.P., Lai L.M.S., Leong L.C.H. (2022). Shear wave versus strain elastography of breast lesions-The value of incorporating boundary tissue assessment. Clin. Imaging.

[10] Gkrozou F., Tsonis O., Daniilidis A., Navrozoglou I., Paschopoulos M. (2021). Tubo-ovarian abscess: Exploring optimal treatment options based on current evidence. J. Endometr. Pelvic Pain Disord..

[11] Goje O., Markwei M., Kollikonda S., Chavan M., Soper D.E. (2021). Outcomes of minimally invasive management of tubo-ovarian abscess: A systematic review. J. Minim. Invasive Gynecol..

[12] Silva F., Castro J., Godinho C., Gonçalves J., Ramalho G., Valente F. (2015). Tratamento minimamente invasivo dos abcessos tubo-ováricos. Rev. Bras. Ginecol. Obstet..

[13] Kakou C., Kasse R., Bakar J. (2024). Transvaginal Echo-Guided Drainage of an Ovarian Tubo Abscess in the Gynecological Emergency of Meulan les Mureaux About a Case and Review of the Literature. Int. J. Reprod. Contracept. Obstet. Gynecol..

[14] Cha S.-W., Kim I.Y., Kim Y.W. (2014). Quantitative measurement of elasticity of the appendix using shear wave elastography in patients with suspected acute appendicitis. PLoS ONE.

[15] Destegül E., Peköz B.Ç., Seyfettinoğlu S., Baş S., Adıgüzel F.I., Narin M. (2023). Ultrasonic shear-wave elastography: A novel method for assessing the tumor grade in endometrial cancer: A prospective study. J. Health Sci. Med..

[16] Acar Sirinoglu H., Uysal G., Nazik H., Destegul E., Okmen F., Pekin O. (2022). Comparison of elasticity values in normal and gestational diabetic pregnancies in the third trimester. J. Obstet. Gynaecol..

[17] Bartolotta T.V., Orlando A.A.M., Dimarco M., Zarcaro C., Ferraro F., Cirino A., Matranga D., Vieni S., Cabibi D. (2022). Diagnostic performance of 2D-shear wave elastography in the diagnosis of breast cancer: A clinical appraisal of cutoff values. Radiol. Medica.

[18] Cosgrove D., Piscaglia F., Bamber J., Bojunga J., Correas J.-M., Gilja O.a., Klauser A., Sporea I., Calliada F., Cantisani V. (2013). EFSUMB guidelines and recommendations on the clinical use of ultrasound elastography. Part 2: Clinical applications. Ultraschall Med.-Eur. J. Ultrasound.

